# Giving Opium

**Published:** 1882-03

**Authors:** 


### GIVING OPIUM.

It is of frequent occurrence that opium and its alkaloids are rejected by the stomach, and the physician is put to his wits' end to find some substitute when hypodermic medication will not do. Owing to the tendency of opium *per orem* to nauseate, belladonna is prescribed; but this is not a perfect substitute, and my experience with Jamaica dogwood leads me to regard it as an unreliable and costly experiment. To overcome the nauseating effect of opium is to break down the principal barrier to the use of a great remedy. To this end I have recently used with unvarying good results the oxalate of cerium in combination. I generally combine one grain of opium with five to ten grains of oxalate of cerium, and have yet to see it rejected by the stomach. It will be useless to give less than five or ten grains of the oxalate.—*Louisville Med. News.*

